# 多发肺转移结节^125^I粒子植入技巧及临床应用

**DOI:** 10.3779/j.issn.1009-3419.2010.03.08

**Published:** 2010-03-20

**Authors:** 卫 李, 刚 但, 建青 姜, 烈 杨, 学全 黄

**Affiliations:** 1 610083 成都，成都军区总医院胸外科 Department of Thoracic Surgery, General Hospital of Chengdu Command, Chengdu 610083, China; 2 400038重庆，第三军医大学西南医院放射科 Department of Radiology, Southwest Hospital, the Third Military Medical University, Chongqing 400038, China

**Keywords:** 肺肿瘤, 经皮穿刺, 近距离放射疗法, 碘放射性同位素, Lung neoplasms, Percutaneous, Brachytherapy, Iodine radioisotopes

## Abstract

**背景与目的:**

^125^I粒子植入治疗肺转移瘤，常因胸部结构复杂，而被视为禁区。本研究旨在探讨CT引导^125^I粒子组织间植入治疗多发肺转移瘤的技术方法和疗效。

**方法:**

在CT引导下对30例患者的115个肺转移灶，行肿瘤内^125^I粒子植入。据多发肺转移瘤的不同位置，肺门部、周围性、胸廓骨骼遮挡肺小结节病变采用相应穿刺植入方法，并行疗效评价。

**结果:**

一次穿刺成功为84.3%（97/115），粒子分布均匀。术后一周复查补种成功为15.7%（18/115）。随访6-24个月，平均14.6个月，CT复查115个病灶中结节完全缓解（complete response, CR）80个，部分缓解（partial response, PR）20个，无变化（No change, NC）8个，疾病进展（progressive disease, PD）7个，总有效率为86.9%。1年局部控制率为93.9%（108/115）。围手术期无严重并发症。

**结论:**

CT引导下采用不同穿刺方法植入放射性^125^I粒子治疗多发肺转移瘤安全微创，并发症发生率低，疗效肯定，是一种微创治疗方法。

## Abstract (en)

**Background and objective:**

^125^I seed implantation treatment of pulmonary metastases, often because of complicated structure of the chest, was considered as restricted areas. The aim of this study is to evaluate the effectiveness of CT guided radioactive ^125^I seed implantation in treating lung metastatic tumors.

**Methods:**

Totally 115 metastatic masses were found in 30 patients with CT guiding. ^125^I seeds were implanted into lung metastatic masses. The approach of implantation was determined according to the location of the lesions (hilar masses, peripheral masses and masses covered by skeletal thorax). The therapeutic effects were evaluated by CT.

**Results:**

Uniform distribution were achieved by single puncture in 84.3% of patients. The rest patients (15.7%) were received replanting. The follow-up period was 6-24 months (mean 14.6). Among 115 lesions in 30 cases, complete response (CR) was achieved in 80 nodes, partial response (PR) in 20 nodes, No change (NC) in 8 nodes and progressive disease (PD) in 7 nodes. The total response rate was 86.9%. The one-year local control rate were 93.9% (108/115). No severe perioperative complications occurred.

**Conclusion:**

CT guided radioactive seed ^125^I implantation is a safe and effective procedure in treating multiple lung metastatic tumor with minimal invasion.

肺转移性小结节病变，紧贴心脏、大血管等重要脏器，通常列为穿刺禁忌。而采用外照射常因剂量受限，效果不佳。而对CT引导穿刺植入手术方法的报道较少。本文是总结30例直径≤2 cm肺内大血管旁小病灶穿刺植入^125^I粒子方法总结，旨在评价此技术的临床意义及安全性。

## 1 资料与方法

### 1.1 一般资料

回顾性分析2006年9月-2008年3月收治的30例胸部转移灶肺癌患者。预计生存期 > 6个月。男18例，女12例，年龄44岁-82岁，平均68.56岁。所有患者均有明确病理诊断，利用TPS计划系统精确制定剂量曲线和放射粒子计划，在CT下将^125^I粒子植入到预定位置。^125^I粒子植入点15 mm-20 mm。处方剂量110 Gy-140 Gy，植入活度为29.6 MBq（0.8 mCi）的粒子3粒-6粒。植入后采用CT影像学进行疗效评价。

### 1.2 手术方法

术前根据CT增强和平扫情况，详细制订穿刺计划，确定穿刺点、穿刺深度。根据增强扫描病灶增强特点避开重要器官、组织以及囊性变区域，以提高穿刺植入治疗的准确率。穿刺前所有患者均行出凝血时间测定，训练患者呼吸并交待注意事项。

#### 1.2.1 肺门部小结节病灶穿刺

操作要点：患者取仰卧或俯卧位，术前增强扫描，了解病灶与血管、支气管的关系，避开重要血管和气管组织确定穿刺路径。肺门部细小结节（直径 < 2.0 cm）的穿刺常用方法：利用CT透视综合了超声的实时和CT的高分辨率、不受气体和骨骼干扰等优点。CT透视法（CT fluoroscopy, CTF）有两种操作方法，分为连续法，持续透视扫描过程中快速进针到预定位置；以及间断法，在扫描间隙内进行穿刺，可以避免不必要的X射线辐射，如靠近肺门部位小病灶的穿刺，用常规CT扫描与CT透视相结合，紧邻血管的病灶使用穿刺成功后甚至需要再次增强了解针槽与肺门血管的关系，防止血管壁受损（图 1A）。紧贴心脏、大血管的小结病灶穿刺易导致上述结构的损伤，常列为穿刺禁忌。通常使用切线依托针尖离去法（图 1B）。即穿刺上述结构边缘小结节时，穿刺针以切线方向接近病灶，针尖斜面朝向器官从而使针尖处于离开方向，确保穿刺安全。

**1:**
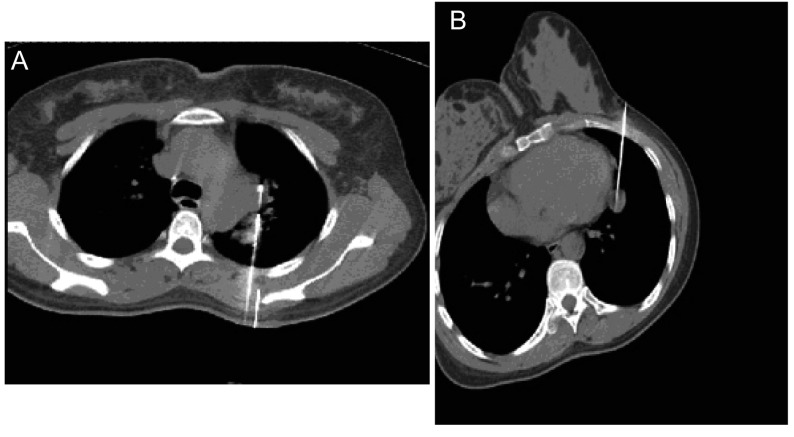
肺门部穿刺 Acupuncture in hilum of lung

#### 1.2.2 周围性肺转移性小结节

肺部小结节指直径 < 2 cm的病灶，尤其下肺野由于下肺呼吸动度大，小结节相应较大幅度运动，加上靶点小更加大穿刺难度。术前与患者沟通消除紧张情绪，训练平静呼吸，争取患者配合，必要时使用镇静剂。在平静呼吸状态下呼气末进针，穿刺到病灶边缘抵住病灶，迅速进针（图 2）。使用CT透视可使穿刺准确、安全（图 3）。

**2:**
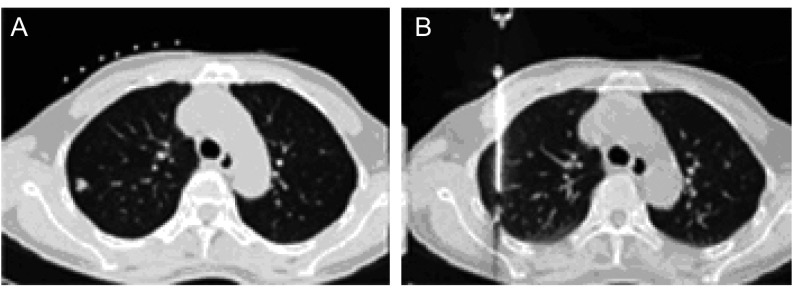
66岁男性，肺鳞状细胞癌，右上肺后段直径约1.0 cm小结节影 66 years old male, lung squamous cell cancer, 1.0 cm tubercle in posterior segment of right upper lung

**3:**
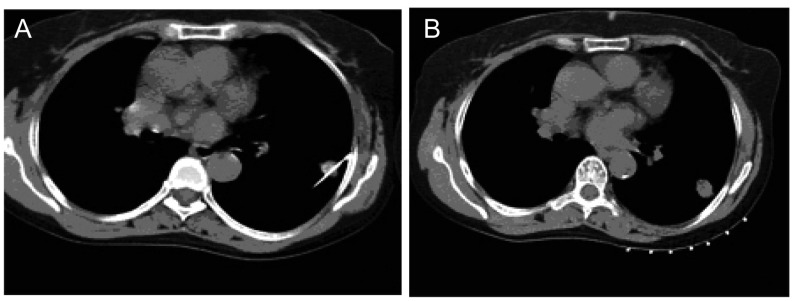
59岁女性，左下肺细支气管肺泡癌 59 years old female, bronchioalveolar carcinoma in left lower lung

#### 1.2.3 胸廓骨骼遮挡肺小结节病变的穿刺

肺部分小结节病灶于垂直穿刺路径会遇到骨骼遮挡，对进针穿刺病灶最佳层面有肋骨、肩胛骨等障碍且不能通过体位改变避开时，可采用倾斜迂曲法穿刺（图 4）。透过肋间隙、上下及左右方向调整针尖方向，倾斜角度穿刺避开肋骨找到最佳针道进针到病灶，对于肩胛骨下病灶从肩胛骨边缘倾斜进针抵达病灶；对于不能避开骨骼穿刺的病灶可直接穿透肋骨、肩胛骨到达其下部病灶，垂直穿刺操作路径短，可避免反复穿刺或变换角度导致的气胸等其它高风险^[1]^（图 5）。调整肋骨或肩胛骨位置，通过上抬双上肢使肋骨呈水平走行相对增大肋间隙，肩胛骨于外旋位置拓宽穿刺路径，此类方法对于部分病灶可以达到目的，应于术前拟定方案并权衡其利弊。

**4:**
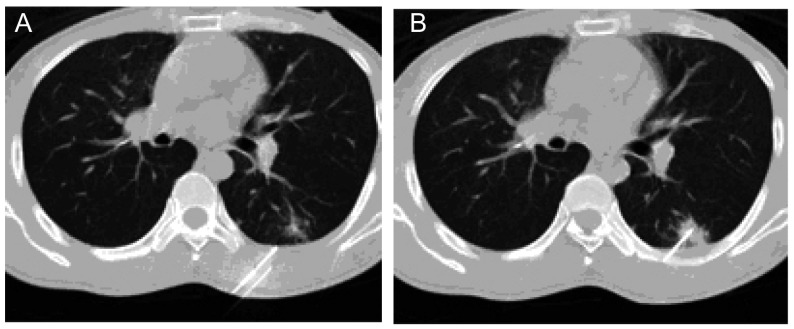
50岁男性，肺腺癌 50 years old male, adenocarcinoma of lung

**5:**
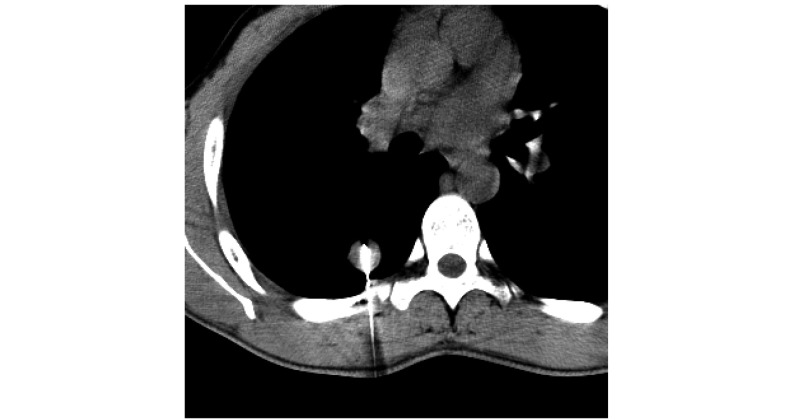
34岁男性，右下肺癌，右肺背段近胸膜下小结节，CT引导下穿肋骨直达病灶穿刺 34-year old male, lung cancer tubercle in right lower lung, CT guided acupuncture directly to target through the ribs

### 1.3 判定标准

所有患者治疗后1月复查CT或MRI，按WHO疗效标准，完全缓解（complete response, CR）：肿瘤完全消失4周以上；部分缓解（partial response, PR）：肿瘤缩小50%，4周以上；无变化（No change, NC）：肿瘤缩小或增大25%以上或出现新的病灶（progressive desease, PD），CR+PR为通过治疗有效。

## 2 结果

### 2.1 置粒情况

病人治疗过程顺利，无严重影响病人生命的并发症，全组未发生粒子误植、胸内大出血及大量气胸等严重并发症。粒子迁移多发生于植粒后数周内，迁移数量较小。

### 2.2 随访与近期疗效

^125^I综合治疗组配对资料显示术后肿瘤大小术后1月行CT检查较术前均有明显变化，肿瘤结节明显缩小，粒子分布良好1个月时（图 6，图 7），CT复查115个病灶中结节CR 80个，PR 20个，NC 8个，PD 7个，总有效率为86.9%，1年局部控制率为93.9%（108/115）。

**6:**
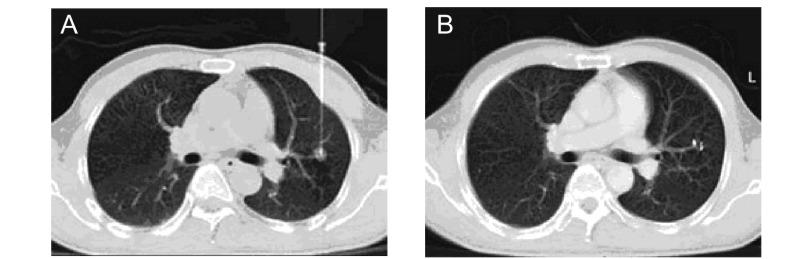
53岁男性肺癌肝转移 53-year old male, lung cancer with hepatic metastasis

**7:**
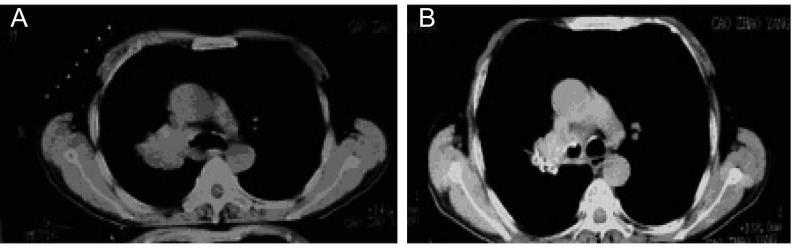
58岁男性，右上肺门部肺癌结节 57-year old male, lung cancer tubercle in hilun of right upper lung, pathologic diagnosis was small cell lung cancer

## 3 讨论

肺转移性小结节穿刺治疗总的原则是术前准确CT增强扫描，制定最佳路径，避免损伤血管；减少穿破胸部重要结构的风险^[2]^。针对肺部转移性肿瘤不同部位病变，穿刺技巧与临床应用改进。

### 3.1 肺门部小结节

肺门部解剖结构复杂，可以根据病变部位采用不同的体位。病变多位于血管间隙内，紧贴心脏、大血管的病灶，穿刺易导致上述结构的损伤，我们在穿刺中多采用CT透视法结合针尖离去法，在保证安全的前提下，兼顾穿刺的准确率^[3]^，在CT透视引导下逐步穿刺到病灶，避免在穿刺过程中伤及心脏、大血管。植入时要考虑到心脏搏动情况。我们认为肺门部病灶位置深，结构复杂，穿刺针道长，因CT透视下穿刺针的针尖可被准确控制，进针更安全准确，还可减少了操作者经验对穿刺的影响，减少穿刺次数和时间，明显提高了穿刺的成功率^[4]^。间断使用CT透视法，可减少患者辐射量吸收。注意要求患者制动，均匀呼吸以减小肺随呼吸移动幅度；临近气管支气管的病变在穿刺前运用中枢性镇咳药物，避免术中刺激导致的咳嗽；术后及时复查有无并发症，并于24 h内紧密随访观察。

### 3.2 周围性肺转移性小结节

肺部周围性转移性小结节的病灶，尤其下肺野由于下肺呼吸动度大，小结节相应较大幅度运动，加上靶点小更加大穿刺难度。术前与患者沟通消除紧张情绪，训练平静呼吸，争取患者配合，必要时使用镇静剂。在平静呼吸状态下呼气末进针，穿刺到病灶边缘抵住病灶，迅速进针。也可使用CT透视，针尖刺入病灶后可在中心部位植入粒子，一般在其周围1 cm距离内也可产生很好的效果。屏气状态下取出穿刺针，减小气胸等并发症。反复穿刺是术后气胸的主要原因。当有大量胸腔积液时，最好先将胸腔积液大部分抽出，暂留置少许胸水以利于壁层和脏层胸膜分隔开，避免肺小结节病灶随积液摆动，要明确穿刺针刺入病变。

### 3.3 胸廓骨骼遮挡肺小结节病变的穿刺

肺部转移性小结节常被胸廓骨骼遮挡。通常CT扫描制定好最佳穿刺路径，根据进针深度及角度在有限的狭小间隙内进针到达病灶即可。对于不能避开骨骼穿刺的病灶可直接穿透肋骨、肩胛骨到达其下部病灶，可用共轴针法，将工作针插入导引针管内送到目的区，进行植入治疗。避免反复穿刺或变换角度导致的气胸等其它高风险，减少并发症，而且共轴针法也可以减少肿瘤细胞从针道种植转移的机率^[5]^。对于位于肩胛骨下的后纵隔部位病灶，可采用肩胛骨于外旋位置，拓宽穿刺路径，此类方法可以到达病变部位^[6]^，但应于术前拟定方案并权衡其利弊。

^125^I粒子治疗对肿瘤复发具有明显疗效，转移性肺癌再手术并发症可高达43.5%。且对化疗不敏感，外照射复发患者再行外照射常效果不佳，多低于60%^[7]^。而对原发灶和转移灶控制得越早，不仅直接减轻相对应部位的危害，而且可减轻或延缓进一步转移的时间和程度，从而延长中位生存时间。本研究对外照射复发患者仍然有效，以及双肺转移病变效果明显，避免了外照射因呼吸动度影响，常扩大2 cm-3 cm照射范围，损伤患者肺脏组织较多弊端。且治疗创伤小，时间短，2天-3天完成，肺复发转移性病变多无手术指针。而外照射因其剂量受限，处方剂量多小于70 Gy^[8]^。剂量过高往往造成严重的皮肤损伤以及纵隔内重要脏器的损伤，且为分次间隔治疗，导致治疗周期一般在5周-7周。局部控制率效果不佳。

本组30例肺转移瘤患者，粒子植入治疗多数一次完成，且每次治疗时间短，处方剂量可达140 Gy。可认为^125^I粒子植入治疗有着不可比拟的优势。控制肿瘤生长总有效率达84.3%。1年局部控制率可达93.9%（108/115）。^125^I粒子治疗纵隔肿瘤安全微创，并发症发生率低，放射性粒子治疗肺癌方法，提高了肿瘤杀伤的疗效，延长患者中位生存时间并弥补了化疗和常规外放疗的不足之处^[9]^。

已有^125^I粒子治疗肺恶性肿瘤的实施，鲜有对穿刺植入技术的报道。由于影像学手段进步，以及在穿刺方法学的不断改进，CT引导下穿刺植入肺复发性小结节病变的，已逐渐被广泛应用于临床，且被证实为切实可行、准确率高、并发症少、近期疗效显著的特点，患者易于接受，值得推广应用。

（本文部分病例资料及图片来自第三军医大学西南医院）
